# Quantification of Immunohistochemically Stained Cells in Skin Biopsies

**DOI:** 10.3390/dermatopathology9020011

**Published:** 2022-03-24

**Authors:** Thomas Emmanuel, Mikkel Bo Brent, Lars Iversen, Claus Johansen

**Affiliations:** 1 Department of Dermatology, Aarhus University Hospital, DK-8200 Aarhus, Denmark; lars.iversen@clin.au.dk (L.I.); claus.johansen@clin.au.dk (C.J.); 2 Department of Biomedicine, Aarhus University, DK-8000 Aarhus, Denmark; mbb@biomed.au.dk

**Keywords:** immunostaining, immunohistochemistry, skin, cells, psoriasis, Adobe Photoshop, QuPath, quantitation, inflammation

## Abstract

Immunohistochemical quantification of inflammatory cells in skin biopsies is a valuable tool for diagnosing skin diseases and assessing treatment response. The quantification of individual cells in biopsies is time-consuming, tedious, and difficult. In this study, we presented and compared two methods for the quantification of CD8^+^ T cells in skin biopsies from patients with psoriasis using both commercial software (Adobe Photoshop) and open-source software (Qupath). In addition, we provided a detailed, step-by-step description of both methods. The methods are scalable by replacing the CD8 antibody with other antibodies to target different cells. Moreover, we investigated the correlation between quantifying CD8^+^ cells normalized to area or epidermal length and cell classifications, compared cell classifications in QuPath with threshold classifications in Photoshop, and analyzed the impact of data normalization to epidermal length or area on inflammatory cell densities in skin biopsies from patients with psoriasis. We found a satisfactory correlation between normalizing data to epidermal length and area for psoriasis skin. However, when non-lesional and lesional skin samples were compared, a significant underestimation of inflammatory cell density was found when data were normalized to area instead of epidermal length. Finally, Bland–Altman plots comparing Qupath and Photoshop to quantify inflammatory cell density demonstrated a good agreement between the two methods.

## 1. Introduction

The quantification of inflammatory cells in skin biopsies is an invaluable instrument for diagnosing diseases and assessing treatment response. Many skin diseases such as hidradenitis suppurative, atopic dermatitis, and psoriasis are dominated by an abundant assortment of immune cells [1,2,3]. Psoriasis is a chronic inflammatory skin disease characterized by epidermal thickening (acanthosis) and the epidermal and dermal infiltration of inflammatory cells, such as CD8^+^ T cells [4]. The disease manifests as recurrent, non-random cutaneous flare-ups [5]. CD8^+^ T cells are thought to be key orchestrators of the underlying inflammation observed in psoriasis. However, other inflammatory cells, including CD4^+^ T cells, neutrophils, macrophages, dendritic cells, and tissue-resident memory T cells, are also involved [6,7,8].

Many methods have been described to quantify the amount of cells in skin biopsies [9]. These methods range from manually counting each individual cell inside one or several semi-randomly selected regions of interest (ROIs) to advanced multi-site artificial intelligence (AI)-guided cell counting [10,11,12,13,14,15,16]. Even though manual cell counting is useful, it is time-consuming when many cells of interest are present [17]. Moreover, considerable heterogeneity exists in how target cells in skin biopsies are quantified and reported.

In this study, we described and discussed two easy-to-use, step-by-step approaches using Adobe Photoshop and Qupath to quantify immune cells, such as CD8^+^ T cells, in skin biopsies. The methods described are scalable to quantify other inflammatory cells by replacing the CD8^+^ antibody with antibodies targeting other antigens in the skin. In addition, we investigated the correlation between quantifying CD8^+^ T cells normalized to area or epidermal length and cell classification using QuPath compared with threshold classification in Photoshop. Finally, we quantified the impact of normalizing inflammatory cells to epidermal length or area on inflammatory cell density in both non-lesional (NL) and lesional skin (LS).

In conclusion, two easy-to-use and scalable methods to rapidly quantify cell densities using cell counts and stained cell area were described. To avoid biases in cell density measurements, we suggest reporting the results normalized to both area and epidermal length.

## 2. Materials and Methods

### 2.1. Skin Biopsies and Sample Preparation

**Figure 1:**
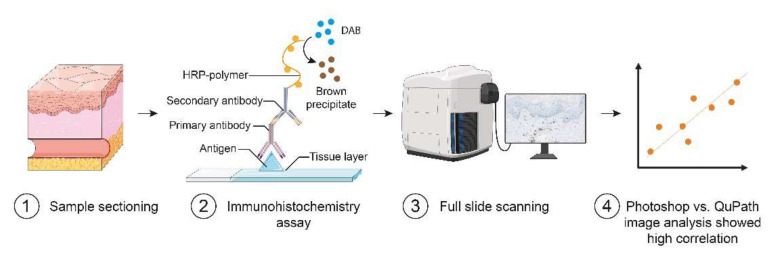
Immunohistochemistry staining and analysis workflow. **①** Skin punch biopsies were obtained from patients with psoriasis at non-lesional (NL) and lesional (LS) sites. **②** A 2,4-Diaminobutyric acid (DAB) immunohistochemistry assay was used to identify CD8^+^ cells. **③** The sections were digitalized using a whole-slide digital pathology scanner. **④** Agreements between inflammatory cell densities estimated using Photoshop and QuPath were assessed using Bland–Altman plots. Created with images from Servier Medical Art (smart.servier.com) and Biorender.com.

Full-thickness skin punch biopsies (23 mm) were acquired from lesional psoriasis plaques (*n* = 10) and peri-lesional unaffected areas (*n* = 10) from ten psoriasis patients. The samples were formalin-fixed and paraffin-embedded. For immunohistochemistry, 4 µm thick tissues were cut on a microtome, placed on slides, deparaffinized, and rehydrated in graded ethanols. Antigen unmasking was performed using heated TEG buffer (TRIS (10 mM) 1.21 g/L, EGTA (0.5 mM) 0.19 g/L, pH = 9.0) at sub-boiling temperature for 20 min. We utilized the 2,4-Diaminobutyric acid (DAB) assay protocol as recommended by the manufacturer to stain CD8^+^ cells. The primary antibody was incubated for 20 min at a concentration of 1:500. The slides were subsequently digitalized using a digital slide scanner (NanoZoomer 2.0-HT; Hamamatsu Photonics K.K., Hamamatsu, Japan) (Figure 1).

### 2.2. Resource Availability

- CD8 (SP16) Rabbit Monoclonal Antibody (Cell Marque, Rocklin, CA, USA) (https://www.cellmarque.com/antibodies/CM/2102/CD8_SP16 (accessed on 30 December 2021)).
- Epredia DAB Quanto Detection System, or any other suitable staining method (https://www.fishersci.dk/shop/products/dab-quanto-chromogen-substrate-2/12693967 (accessed on 30 December 2021)).
- NanoZoomer 2.0-HT; Hamamatsu Photonics K.K. (Hamamatsu, Japan) (https://nanozoomer.hamamatsu.com/jp/en/index.html (accessed on 30 December 2021)).
- NDP.view2 (Hamamatsu Photonics K.K., Hamamatsu, Japan) (https://www.hamamatsu.com/eu/en/product/type/U12388-01/index.html (accessed on 30 December 2021)).
- Adobe Photoshop 2021 (https://www.adobe.com/products/photoshop.html (accessed on 30 December 2021)).
- QuPath v. 0.3.0 (https://qupath.github.io/ (accessed on 30 December 2021)) [18].

### 2.3. Step-By-Step Guide

#### 2.3.1. Convert NDPI to TIFF

1. Open NDP.view2 and select scanned slide (.ndpi).
2. Use the *Rotate Widget* in the right-side panel to rotate the slide and align it horizontally.
3. Zoom to ensure the whole epidermis and a part of the dermis are visible.
4. Right-click to select *Export* → *Export Image* (Ctrl + E).
5. Save as .tif using ×20 lens and 300 DPI.

#### 2.3.2. Adobe Photoshop: Regions of Interest

**Figure 2:**
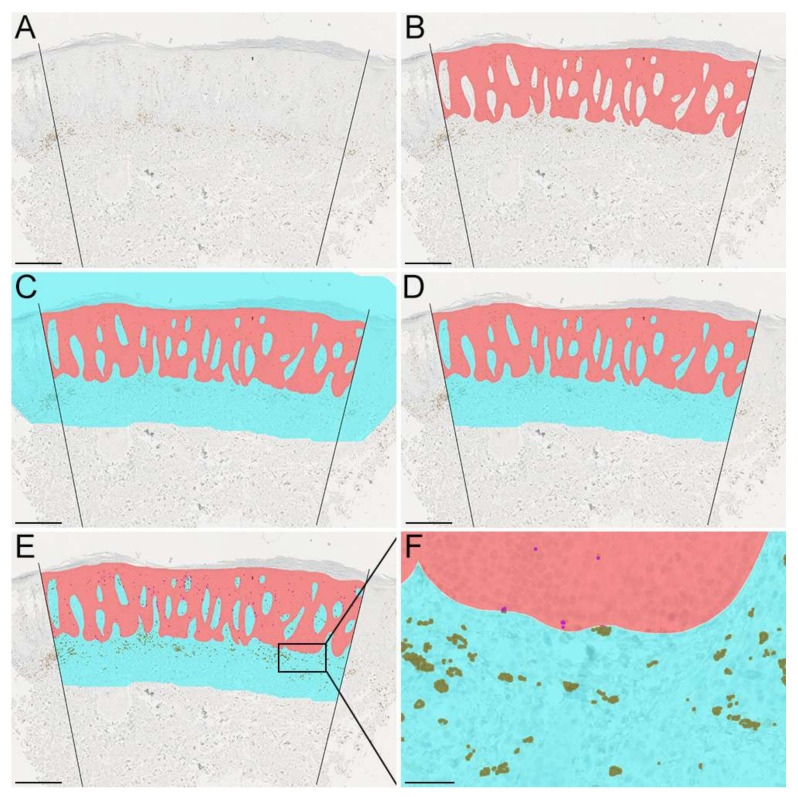
Photoshop workflow. (**A**) Demarcation of the borders of the region of interest (ROI). (**B**) Creation of an epidermal ROI (red). (**C**) Expansion of the epidermal ROI creating a dermal ROI (cyan). (**D**) Deletion of areas on the apical side of the epidermis and outside the demarcated borders. (**E**) Painting of CD8^+^ stained cells. (**F**) Magnification of the area depicted on (**E**) stains (brown: CD8^+^ stained cells in dermis. Purple: CD8^+^ stained cells in the epidermis). (**A**–**E**): Scale bar = 400 µm. (**F**): Scale bar = 50 µm.

1. Use the *Line tool* (U) to make two large lines placed with both sides orthogonal to the apical part of the epidermis (Figure 2A).
2. Use the *Brush tool* with a 100% hardness to manually demarcate the epidermis and use one color for the whole epidermis (Figure 2B).
3. Click *Select* → *Color Range* to select color of epidermis. Click *Ok.*
4. Use the right-side panel to select *Histogram* →, select *Expanded View* →, click *Uncaged Refresh* (refresh symbol on the right side). The number of pixels is now shown for the epidermal area.
5. Go to *Image* *→ Analysis* *→ Ruler Tool* to measure the scale bar. L1 denotes the number of pixels corresponding to the scale bar’s distance.
6. Go to *Select Image* *→ Analysis* *→ Set Measurement Scale* *→ Custom* (input the length of the scale bar in pixels). In this example, 100 µm corresponds to 220 pixels.
7. *Select→ Modify µm Expand* to expand the selected epidermal region by 400 µm (corresponds to 880 pixels). Expand the region twice by 440 pixels because Photoshop does not allow expansions above 500 pixels.
8. Create a new layer (Ctrl + Shift + N), name it “dermis”, and fill the layer with a new color (*Edit* *→ Fill* or press Shift + F5) (Figure 2C).
9. Go to the epidermal layer, and on the left-side panel, select the *Magic Wand Tool*. Click on the epidermis to select this layer and go to the dermis layer to remove the selection from that layer. Manually delete areas at the apical part of the epidermis and outside the boundaries of the two demarcated lines (Figure 2D).
10. Finally, the epidermal length is estimated. There is no easy way to calculate the length of a polygonal line in Photoshop. We recommend using Adobe Illustrator or QuPath to do this (see the section about QuPath below). However, it is possible to estimate the epidermal length in Photoshop by repeated measures of small straight lines. Go to *Image* *→ Analysis* *→ Ruler Tool* and open *Window* *→ Measurement Log* to outline repeated lengths. After each outline, press *Record Measurements*. The repeated measures of the small straight lines can be added to estimate the total epidermal length.

#### 2.3.3. Adobe Photoshop: Threshold Classification

1. Next, the stained cell area is calculated. Open the .tif file.
2. *Select → Color Range* and hold Shift to select multiple colors of target cells. Click *Ok.*
3. *Select → Modify → Expand* and expand the selected regions by 2 to 3 pixels to make the selection more coherent.
4. Create a new layer. *Layer → New → Layer* (Shift + Ctrl + N). Name the new layer “total cell area”.
5. *Edit → Fill* (Shift + F5) and choose the brown color.
6. Manually erase obvious misclassifications of stained cells.
7. Make two duplicates of the layer “total cell area” and name the two new layers “epidermal cell area” and “dermal cell area”.
8. Select the “dermis” layer and click *Select → Color Range* to select the color of dermis. Click *Ok.*
9. Select the “dermal cell area” layer and right-click on the selection and press *Select Inverse* and press *Delete*. Now, all cells in the dermal region of interest remain.
10. Repeat steps 8 and 9 but replace “dermis” with “epidermis”.
11. For both the dermal and epidermal layer independently: *Select → Color Range* and click on the brown color. Use the right-side panel to select *Histogram → Select Expanded View* → and click *Uncaged Refresh* (refresh symbol on the right side). Now, the number of pixels of the selection can be shown (epidermal cell area for the “epidermal cell area” layer and “dermal cell area” layer) (Figure 2E,F).

#### 2.3.4. Adobe Photoshop: Cell Counting

1. Sometimes, the circularity and number of cells makes it very easy to count instead of measuring cell area. However, in cases with many cells, it might be time-consuming.
2. Click on Image → Analysis → Count Tool.
3. Click on individual cells in the epidermis and dermis ROIs to count.

#### 2.3.5. QuPath: Cell Counting

1. In the top pane, select *Tools → Points* to count individual cells.
2. Randomizer.org can be used if counting from randomly selected grids inside ROIs is needed.
3. In the top pane, press *Show Grid.*

#### 2.3.6. QuPath: Create Training Annotations

**Figure 3:**
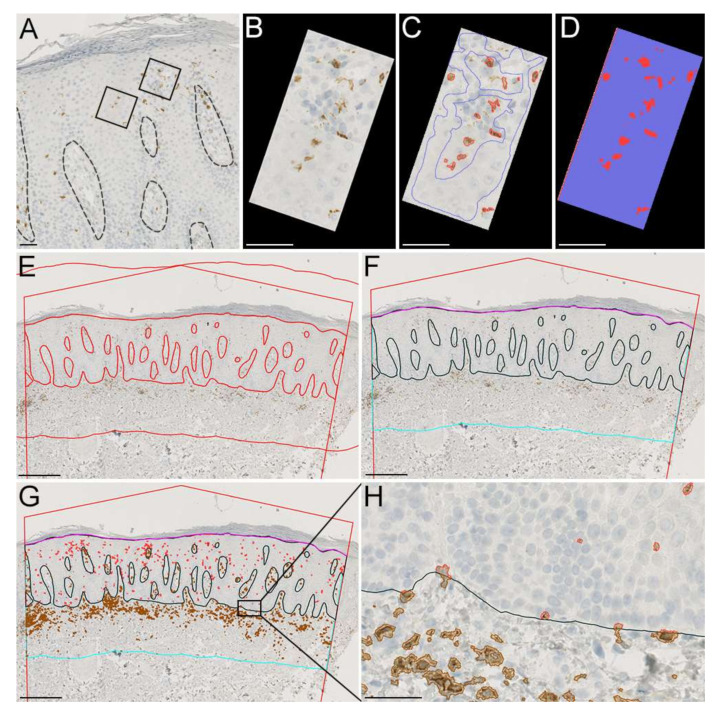
QuPath workflow. (**A**–**D**) Example of training images with CD8^+^ stained cells (red) and areas that should be excluded (blue). (**E**) Demarcation of the borders of the regions of interest (ROIs) with an expansion of the epidermal ROI creating a dermal ROI. (**F**) Recoloring of ROIs and deletion of areas on the apical side of the epidermis and outside the demarcated borders. (**G**) Classification of CD8^+^ stained cells inside the epidermal and dermal ROI. (**H**) Magnification of the area depicted on (**G**) stains (brown: CD8^+^ stained cells in dermis. Red: CD8^+^ stained cells in the epidermis). (**A**,**E**–**G**): Scale bar = 400 µm. (**B**,**D**,**H**): Scale bars = 50 µm.

1. Drag and drop the .ndpi files into the project window.
2. First, a classification application needs to be trained to aid in the classification of stained cells.
3. Click Classify → Training images → Create region annotations → Width 100, Height 100, size units µm. Set Classification Region* → and press Create region.
4. Place training regions with representative areas with cells that should be classified and areas that should be excluded from the classification (Figure 3A).
5. Click *Classify → Training images → Create training image*. Select classification Region* and leave everything else untouched. This will create a new sparsed image combining all training images (Figure 3B).
6. Manually outline each cell staining of interest on the sparsed images and click *Set class → Positive* (or manually create a new class) and representative areas that should not be included (*Set class → Negative*) (Figure 3C).
7. Go to Classify → Pixel classification → Train pixel classifier (Ctrl + shift + P).
8. Use classifier: Artificial neural network (ANN_MLP), resolution very high (0.91 µm/px), and leave everything else as default.
9. Press live prediction (Figure 3D).
10. Classification is an iterative process and might require adjustments; however, once satisfied with the classification, insert the classifier name and press *Save*.

#### 2.3.7. QuPath: Regions of Interest and Running the Classification Application

1. Go to annotations.
2. Make a large, irregular pentagon placed with both sides orthogonal to the apical part of the epidermis (Figure 3E).
3. Use the tools in the upper bar to manually “paint” the epidermis. NB: The Alt key can be used to quickly erase an area.
4. If separate epidermal and dermal area measurements are not warranted, then a precise delineation of the epidermal or dermal compartment is not as important, so long as no visible cells are misclassified in the wrong compartment.
5. To create a dermal compartment, first press the epidermal annotation, then go to *Objects → Annotations → Expand annotations.*
6. In the window, input how much the dermal area should encompass. In our example, CD8^+^ cells are located close to the epidermis, and an area expanding 400 µm below the ventral part of the epidermis selects the vast majority of CD8^+^ cells in the dermis.
7. Press “Remove interior” and press “Run”.
8. A new annotation will appear; however, the area to the left, right, and apical part of the epidermis is not needed. Manually de-select this area using *Tools → Brush.*
9. The epidermal length can be made using the *Tools → Polyline.* Press once and make a line following the apical part of the epidermis inside the pentagon.
10. Select Epidermis and Dermis under Annotations and go to Classify → Pixel classification → Load pixel classifier → Choose model → Classifier.
11. Under “*Region*”, choose ”*Any annotations*” and press “*create objects*” →. Under “Choose parent object”, select “*Current selection*” → and new window will appear. Press *OK* to the default options.
12. A new annotation will now be created containing positive cells in the epidermal and dermal segments (Figure 3G,H).

## 3. Results

### 3.1. Comparison between Inflammatory Cell Density Normalized to Epidermal Length or Area

**Figure 4:**
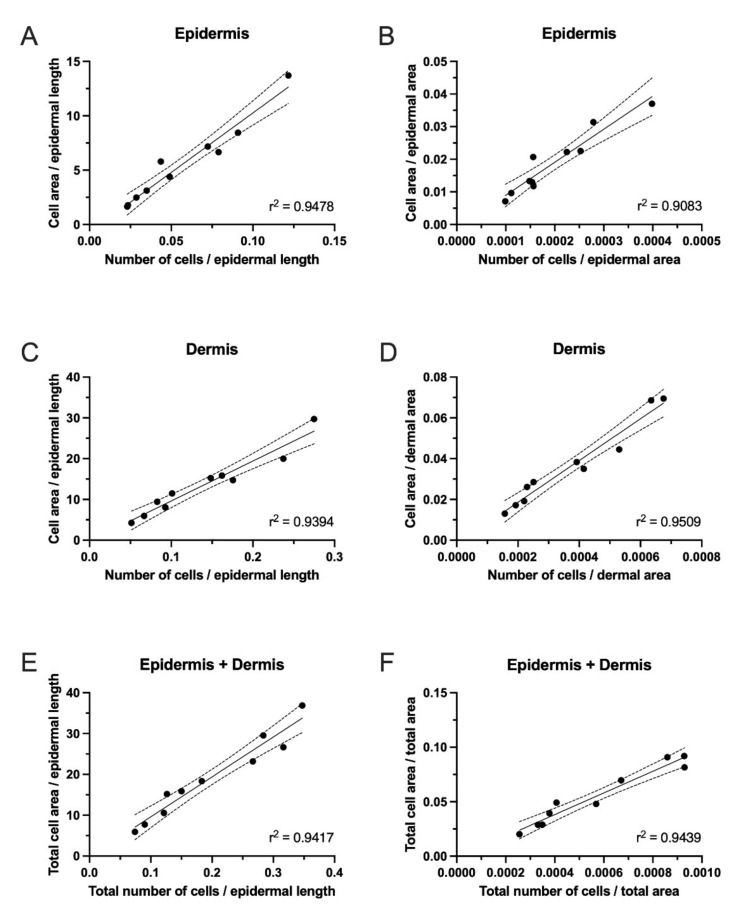
Inflammatory cell density measurements from lesional skin (LS) from ten patients with psoriasis. Linear correlations between results obtained by QuPath analyses of CD8+ cells normalized to length or area. The coefficient of determination (r2) demonstrated a good correlation between quantifying cells using cell area and counts in LS skin. (**A**) Correlation between the cell area in the epidermis normalized to the epidermal length and the number of cells in the epidermis normalized to the epidermal length. (**B**) Correlation between the cell area in the epidermis normalized to the epidermal area and the number of cells in the epidermis normalized to the epidermal area. (**C**) Correlation between the cell area in the dermis normalized to the epidermal length and the number of cells in the dermis normalized to the epidermal length. (**D**) Correlation between the cell area in the dermis normalized to the epidermal area and number of cells in the dermis normalized to the epidermal area. (**E**) Correlation between the total cell area normalized to the epidermal length and the total number of cells normalized to the epidermal length. (**F**) Correlation between the total cell area normalized to the total area and total number of cells normalized to the total area.

Cell counts or cell areas are often used to quantify cells in the skin. However, there is currently no consensus on whether to normalize the results to epidermal length or area. Therefore, we compared the inflammatory cell density in lesional skin assessed by cell counts or cell area normalized to either epidermal length or area. The correlations were assessed using simple linear regression. Correlation analyses were performed for ROIs containing the epidermis (Figure 4A,B), dermis (Figure 4C,D), and epidermis and dermis combined (total) (Figure 4E,F). In general, we observed an acceptable correlation (r^2^ ranging from 0.91 to 0.95) between quantifying cells using cell area and cell counts, suggesting that both methods equally assessed the infiltration of CD8^+^ cells in LS skin.

### 3.2. Comparison of Cell Classification Using QuPath with Threshold Classification Using Photoshop

**Figure 5:**
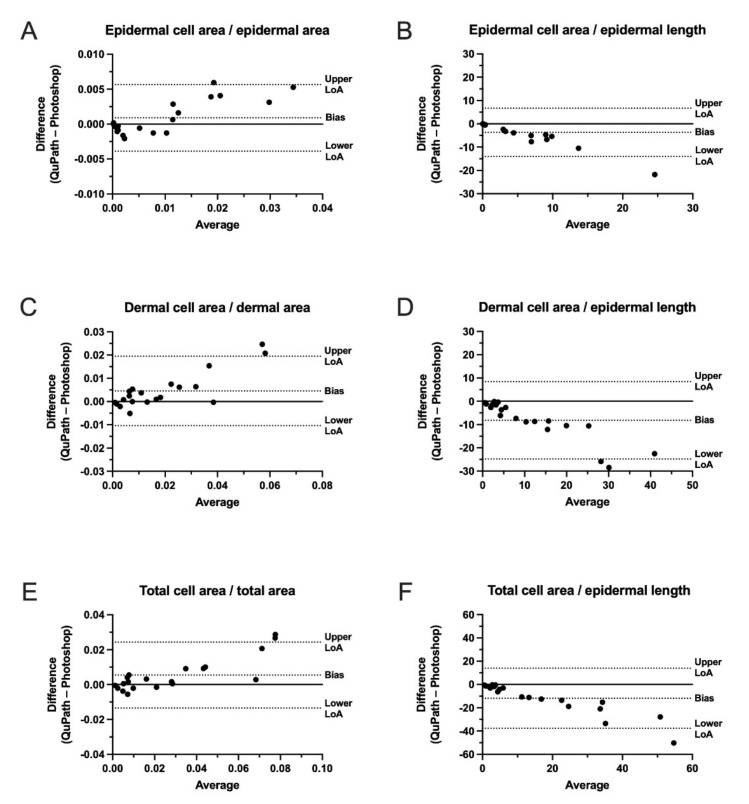
Bland–Altman plots comparing the mean difference between estimations of CD8^+^ cells in the epidermis and dermis normalized to area or epidermal length using QuPath and Photoshop. The total cell area represents the combined cell area of CD8^+^ cells in the epidermal and dermal layers. The total area represents the combined area of the epidermal and dermal layers. (**A**) Comparison between the epidermal cell area and the epidermal area. (**B**) Comparison between the epidermal cell area and the epidermal length. (**C**) Comparison between the dermal cell area and the epidermal area. (**D**) Comparison between the dermal cell area and the epidermal length. (**E**) Comparison between the total cell area and the total area. (**F**) Comparison between the total cell area and the epidermal length. LoA: Limits of Agreement.

We compared cell classifications performed in QuPath with threshold classifications obtained in Photoshop to identify CD8^+^ cells. Bland–Altman plots were used to analyze the agreement between the two methods for ROIs containing epidermis (Figure 5A,B), dermis (Figure 5C,D), and epidermis and dermis combined (total) (Figure 5E,F). A good agreement was found between the two methods. However, with larger inflammatory cell densities, an increased difference between the two methods was observed. This suggests that mixing the two classification methods is not advisable.

### 3.3. Effect of Increased Epidermal Thickness on Inflammatory Cell Density

**Figure 6:**
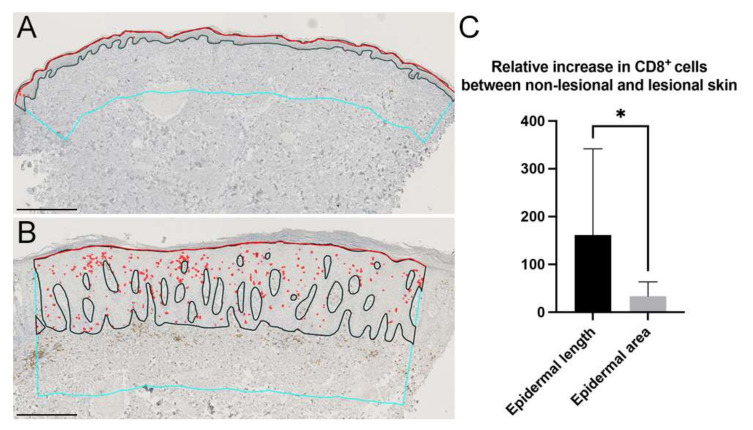
Example of increased epidermal thickness between (**A**) non-lesional skin (NL) and (**B**) lesional skin (LS). Black line: interphase between the dermis and epidermis. Red line: epidermal length. Red: CD8^+^ cells. Cyan lines: demarcation of the dermis, height 400 µm. (**C**) Relative increase in CD8^+^ cells in NL and LS skin normalized to the epidermal length or epidermal area. The relative amount of cells significantly decreased when normalized to the epidermal area compared with epidermal length, reflecting an increased epidermal thickness in LS. Scale bars = 400 µm. Data are presented as mean ± SD. Statistical comparison: Student’s *t*-test, * *p* < 0.05.

When normalizing inflammatory cell densities to the epidermal area, diseases where extensive epidermal thickening is present, such as acanthosis in psoriasis, might “dilute” the estimated cell density due to an increased epidermal area. Therefore, we compared the difference between normalizing the inflammatory cell density to the epidermal area and epidermal length. We used paired NL and LS skin samples (Figure 6A,B) and calculated the relative increase between NL skin and LS skin using cell area normalized to epidermal area or normalized to epidermal length. We found that if data on inflammatory cell densities were normalized to epidermal area rather than epidermal length, the density was decreased by almost 73% (Figure 6C). These findings demonstrate the importance of carefully considering how best to quantify data obtained from skin samples with excessive epidermal thickness. To overcome such “dilution” from increased epidermal thickness, we suggest reporting inflammatory cell densities both normalized to epidermal length and normalized to the epidermal area.

## 4. Discussion

Manual counting is a tedious but sometimes necessary task. However, the labor intensiveness of the procedure might also limit the number of slides that can be analyzed. New image analysis tools have opened up new ways of quantifying cells in the skin [19]. Computerized image analysis is a relatively simple and reproducible form of analysis and is superior in assessing epidermal thickness [20]. In theory, a large amount of data can be easily and rapidly analyzed and quantified reliably, resulting in numerical data that may correlate with biological effects. Using trainable pixel classifiers allows for increased reproducibility and can potentially be iteratively improved with more extensive training sets as previously described [17]. An estimation of the area fraction of stained cells and length fraction of stained cells in the skin can be performed in skin tissue sections and takes full advantage of current image analysis software capabilities.

Many methods have been used to assess the amount of cells in the skin, such as counts per area [11], stained area using threshold values [12], counts per high field area [13], digital analysis of cell numbers on ROIs positioned at high cell concentration areas [14], counting using superimposed grids [15], and raw counts with no normalization [16]. Furthermore, quantifying cells in immunofluorescence, imaging mass cytometry, and digital spatial profiling are widely used [21,22,23].

In this study, we demonstrated two easy-to-use methods to quantify the amount of inflammatory cells in the skin using both commercially available and open-source software. Both methods demonstrated a satisfactory agreement, although their agreement diverged slightly at increasing inflammatory cell densities. Moreover, our results suggested a relatively high correlation between normalizing inflammatory cell densities to epidermal length and area in LS skin. However, we suggest reporting data for cell densities as normalized to both epidermal length and area to overcome a potential “area dilution” of skin samples in diseases with thickened epidermis that leads to underestimating the inflammatory cell quantity.

Caution should be taken when assessing cells in a confined area using an area fraction. Although speculative, one might experience a scenario in which the absolute cell count in a given area is increased, but because the epidermal area also increases, the cell count as a fraction of the area might actually decrease. Normalizing data to the epidermal length solves this issue and allows for easier comparison across studies. This is especially important when estimating inflammatory cell densities in the dermal compartment as most inflammatory cells are located in the apical part close to the epidermal interphase. If a too-large dermal ROI is used, the inflammatory cell density might be underestimated.

The methods described have some limitations inherent to the histological assessment of cells. Larger and rounder cells have a greater probability of being counted [24]. High-quality histological sections are necessary to correctly classify differences in the colors of target cells (e.g., CD8^+^ T cells) and non-target cells (e.g., the remaining cells present in the skin). Although QuPath is open-source, it requires digitalized histological sections, which might be an obstacle when slide scanners are not readily available. Furthermore, using cell counts to measure the quantity of Langerhans cells or cells with a dendritic morphology might cause an overestimation of cell densities. Finally, melanin produced from sun-exposed skin might directly influence the correct counting of positively stained cells in the skin. Even though unsupervised analysis is possible, it will almost always be necessary to perform manual corrections on images after performing staining classification.

**Figure 7:**
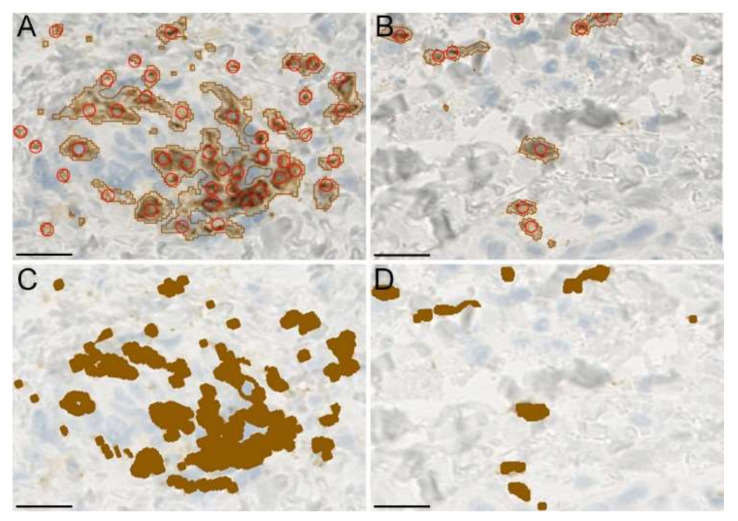
(**A**,**B**) Example of cell classification in high-density and low-density infiltrates using QuPath and manual counting (red circles). (**C**,**D**) Classification of infiltrates using Photoshop. Scale bars = 20 µm.

Differences in inflammatory cell densities are widely present in NL- and LS-psoriasis skin. Although it cannot be precluded that this affects the results obtained with the methods demonstrated in this study, we found a satisfactory visual agreement between classifications of cells in high-density infiltrates and low-density infiltrates using QuPath and Photoshop (Figure 7).

The methods demonstrated in this manuscript may be adopted by both pre-clinical and clinical researchers in the field of dermatology. The methods are particularly valuable to the area of dermatopathology, where cell quantifications in the dermal and epidermal area are warranted. However, the methods are also easy to use and scalable to areas where the quantification of immunohistochemically stained cells is needed.

Semi-automated image analysis of skin samples can, in theory, provide reproducible and unbiased numerical estimations of the amount of cell labeling. However, none of the methods can correct for possible variations among different staining intensities on the samples. Therefore, caution must be exercised to ensure a high standardization of staining.

## 5. Conclusions

The quantification of target cells in the skin is reported in various ways, making comparisons between studies difficult. We provided two easy-to-use and scalable methods to rapidly quantify cell densities using cell counts and stained cell area, and we suggest reporting the results normalized to both area and epidermal length.

## Data Availability

The raw data supporting the conclusions of this article are available by the authors upon reasonable request.
